# Circulating endothelial cells and angiogenic serum factors during neoadjuvant chemotherapy of primary breast cancer

**DOI:** 10.1038/sj.bjc.6602952

**Published:** 2006-01-31

**Authors:** G Fürstenberger, R von Moos, R Lucas, B Thürlimann, H-J Senn, J Hamacher, E-M Boneberg

**Affiliations:** 1 Center for Tumor Detection and Prevention, Rorschacherstrasse 150, 9006 St Gallen, Switzerland; 2 Senology Center of Eastern Switzerland, 9006 St Gallen, Switzerland; 3 Biochemical Pharmacology, University of Konstanz, 78464 Konstanz, Germany; 4 Internal Medicine V/Pulmonary Division, University Hospital Homburg, 66421 Homburg, Germany; 5 Biotechnology Institute Thurgau, 8274 Tägerwilen, Switzerland

**Keywords:** breast cancer, angiogenesis, chemotherapy, circulating endothelial cells, angiogenic factors

## Abstract

Circulating endothelial cells (CECs) as well as bone-marrow-derived endothelial precursor cells (EPC) play an important role in neovascularisation and tumour growth. To study the impact of neoadjuvant chemotherapy on the amounts of CEC and their precursor cells, mature CEC and their progenitors were quantified by flow cytometry in peripheral blood of breast cancer patients during anthracycline and/or taxane based neoadjuvant chemotherapy and subsequent surgery in comparison to age-matched healthy controls. Cell numbers were tested for correlation with serum levels of angiopoietin-2, erythropoietin, endostatin, endoglin, VEGF and sVCAM-1 as well as clinical and pathological features of breast cancer disease. Circulating endothelial cells were significantly elevated in breast cancer patients and decreased during chemotherapy, whereas EPC (CD34^+^/VEGFR-2^+^) as well as their progenitor cell population CD133^+^/CD34^+^ and the population of CD34^+^ stem cells increased. Concomitantly with the increase of progenitor cells an increase of VEGF, erythropoietin and angiopoietin-2 was observed. These data suggest that chemotherapy can only reduce the amounts of mature CEC, probably reflecting detached cells from tumour vessels, whereas the EPC and their progenitors are mobilised by chemotherapy. Since this mobilisation of EPC may contribute to tumour neovascularisation an early antiangiogenic therapy in combination with chemotherapy could be beneficial for the success of cancer therapy.

Gaining access to the hosts vascular system and the maintenance of sufficient blood supply are growth-limiting steps in tumour progression. Most tumours form endothelial cell based vessels by angiogenesis, the sprouting of new vessels from existing vessels, but also an adapted form of the embryonic process of vasculogenesis can be observed, where blood vessel are formed *de novo* from endothelial precursor cells (EPC). In this process, EPC can be mobilised from the bone marrow, transported through the blood stream to become incorporated into the walls of growing blood vessels (Rafii *et al*, 2002a; Bergers and Benjamin, 2003). Endothelial precursor cells may reflect the phenotype of embryonic angioblasts, which are migratory endothelial cells with capacity to circulate, proliferate and differentiate into mature cells, but have not acquired the characteristics of mature endothelium. Endothelial precursor cells can be mobilised from the bone marrow and recruited to the sites of neoangiogenesis by angiogenic growth factors as VEGF, angiopoietin and erythropoietin (reviewed in Rafii, 2000; Rafii *et al*, 2002b; Bahlmann *et al*, 2004; Ribatti, 2004).

The search for these EPC has shown that a subset of the pluripotent CD34^+^ stem cells has phenotypic features of endothelial cells. The EPC were further analysed to be in the population of CD133^+^/CD34^+^ progenitor cells. In the bone marrow, the early EPC are characterised by their expression of CD34, CD133 and the VEGFR-2 (Flk-1, KDR) (Peichev *et al*, 2000). In the circulation of adults, more mature EPC are found that have lost CD133, but are still positive for VEGFR-2 and CD34 (Rafii, 2000; Hristov *et al*, 2003).

Endothelial precursor cells as well as mature endothelial cells are detectable in the peripheral circulation (Ribatti, 2004). Mature endothelial cells may appear in the circulation by detaching from activated or damaged vessels. Mature endothelial cells express also CD34 and VEGFR-2, but unlike the EPC they are negative for the haematopoietic marker CD45 (Mancuso *et al*, 2001). An increase of circulating endothelial cell (CEC) was described in several pathologic conditions that involve vascular injury or instability as myocardial infarction, infectious vasculitis and cancer (George *et al*, 1993; Mutin *et al*, 1999; Mancuso *et al*, 2001; Beerepoot *et al*, 2004). These CEC are mostly viable and exhibit still proliferative capacity despite their terminal differentiation (Lin *et al*, 2000; Beerepoot *et al*, 2004; Ribatti, 2004).

Antiangiogenic therapy is a promising new form of cancer treatment and the effectiveness of new angiogenesis inhibitors are currently tested in several studies. Also for standard chemotherapy regimen as taxanes and cyclophosphamide antiangiogenic properties are discussed (Belotti *et al*, 1996; Hotchkiss *et al*, 2002; Lennernas *et al*, 2003). An antiangiogenic effect may be reflected by a decrease of CEC or EPC. In this study, we quantified mature CEC and progenitor cells in peripheral blood of breast cancer patients undergoing anthracycline and/or taxane based neoadjuvant treatment and subsequent surgery. The amounts of mature CEC and progenitor cells were correlated with serum levels of angiogenic growth factors as well as classic clinical and pathological features of breast cancer disease.

## PATIENTS AND METHODS

### Patients

The study was approved by the regional ethic board of St Gallen, Switzerland, and all patients and controls gave written informed consent before study entry. We studied 10 female patients (for details see Table 1) with locally advanced primary breast cancer during the course of treatment with neoadjuvant chemotherapy and subsequent surgery (Group A). From these patients, EDTA and serum blood samples were withdrawn at the time of diagnosis, after two cycles of chemotherapy, directly before surgery as well as 1 day and 4 weeks after surgery. Blood samples from age-matched healthy females were analysed accordingly. The mean age±s.e.m. was 49.3±2.6 years (range 38–61 years) for the patients and 49.2±2.5 years (range 37–60 years) for the controls.

Additionally, we analysed blood from 16 female patients (details see Table 1) with primary breast cancer at the time of diagnosis (Group B). Measurement of endothelial cells was carried out within 24 h after venous puncture. For the serum samples, blood was withdrawn in additive-free vacutainers (BD Vacutainer Systems, Basel, Switzerland) and allowed to clot for 30 min at room temperature. Then the samples were centrifuged at 2700 **g** for 10 min, and the serum was aliquoted and stored at −80°C until ELISA measurement.

The amount of endothelial cells and the concentration of serum factors were correlated with the clinical tumour stage and the following pathological features (TNM classification; Greene *et al*, 2002): pathological tumour size (pT, ypT respectively; y stands for histological assessment after antineoplastic therapy), nodal status (pN, ypN respectively), tumour grading, oestrogen receptor status, progesterone receptor status and regression grade after Sinn *et al* (1994). Immunohistochemical expression of HER-2/neu was evaluated according to the published scoring guidelines of the HercepTest (Dako, Zug, Switzerland). Patients were subgrouped according to their immunohistochemistry (IHC) score and the result of the fluorescence *in situ* hybridisation into a FISH positive or IHC score 3+ group *vs* a FISH negative or IHC score 1+ group.

### Measurement of angiogenic factors

Serum levels of growth factors were quantified by sandwich ELISA. FGF basic, VEGF and sVCAM-1 were measured using DuoSet ELISA Kits from R&D Systems (Wiesbaden, Germany), according to the manufacturer's instructions with minor modifications: For sVCAM-1, the capture antibody was used with 0.5 *μ*g ml^−1^ and the tracer with 72 ng ml^−1^, the highest standard was 5000 pg ml^−1^. Soluble endoglin (sCD105), erythropoietin, angiopoietin-2, and endostatin were measured using Quantikine ELISA kit (R&D Systems) according to the manufacturer's instructions.

### Flow cytometry

For measurement of CECs and progenitor cells, a method from Mancuso *et al* (2001) was adapted. Flow cytometric analysis was carried out in whole blood without any enrichment procedure to avoid enrichment artefacts. Mature CEC were defined as negative for haematopoietic marker CD45 and positive for endothelial markers CD146 (P1H12), CD31 and CD34. Activated CEC were defined as CD45^−^, CD34^+^, CD105^+^ or CD106^+^. Endothelial precursor cells were defined as CD34^+^, CD45^low^ and VEGFR-2^+^. For the flow cytometric analysis, 100 *μ*l EDTA blood was stained with one of the following antibody panels: (A) 5 *μ*l anti-CD31-FITC (clone WM59, Serotec), 1 *μ*l P1H12-PE (clone P1H12, Chemicon), 5 *μ*l anti-CD45-PerCP (clone 2D1, BD Biosciences, Allschwil, Switzerland) and 5 *μ*l anti-CD34-APC (clone 8G12, BD Biosciences). (B) 5 *μ*l anti-CD31-FITC, 5 *μ*l anti-CD133-PE (clone AC133, Miltenyi Biotec, Bergisch Gladbach, Germany), 5 *μ*l anti-CD45-PerCP and 5 *μ*l anti-CD34-APC. (C) 5 *μ*l anti-CD106-FITC (clone 1.G11B1, Serotec), 5 *μ*l anti-CD105-PE (clone SN6, Serotec), 5 *μ*l anti-CD45-PerCP and 5 *μ*l anti-CD34-APC. (D) 20 *μ*l anti-KDR-Biotin (goat IgG, R&D Systems, Wiesbaden, Germany), 5 *μ*l anti-CD133-PE, 5 *μ*l anti-CD45-PerCP and 5 *μ*l anti-CD34-APC and after washing with PBS in a second staining step 1 *μ*l Streptavidin-FITC (Dako) was added to the blood and incubated for 15 min at room temperature. For quantification of CECs and progenitor cells, a known amount of 6 *μ*m latex microspheres (Polyscienes, Eppelheim, Germany) was added to the blood as an internal standard. After incubation for 30 min at room temperature, 1 ml BD Lysing Solution (Becton Dickinson) was added to lyse erythrocytes and fixate cells. After 10 min, cells were washed twice with 1 ml PBS and measured in a FACS LSR flow cytometer (BD Biosciences) using Cell Quest software (BD Biosciences). In each blood sample, 300 000 events were counted. Gating details are given in the supplementary online material (Supplementary Figure 1).

### Statistical analysis

Statistical analysis was performed using GraphPad Instat (Instat Statistics, GraphPad Software). For all data parametric testing was performed. For the comparison of the subsequent blood withdrawals from the same patients paired testing with a paired *t*-test was performed. For the comparison of patients and controls or patient subgroups, an unpaired *t*-test was used. Differences were considered statistically significant at *P*<0.05.

## RESULTS

### Quantification of CEC

**Figure 1:**
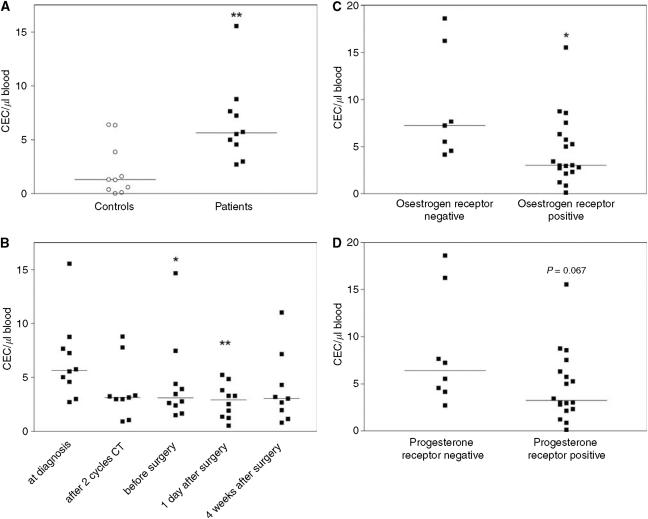
Flow cytometric quantification of CEC. The amounts of CEC were quantified in peripheral blood samples of breast cancer patients and matched controls by four colour flow cytometry analysis. (**A**) Comparison of CEC amounts in blood samples from patients (group A) at the time of diagnosis and matched controls. ^**^P<0.01 *vs* matched controls. (**B**) CEC levels during the course of neoadjuvant chemotherapy and subsequent surgery. ^*^P<0.05, ^**^P<0.01 *vs* values at time of diagnosis. (**C**) Comparison of CEC amounts in blood samples from breast cancer patients (Groups A+B) with oestrogen receptor negative and oestrogen receptor positive tumours at the time of diagnosis. ^*^P<0.05 *vs* values of oestrogen receptor negative patients. (**D**) Comparison of CEC amounts in blood samples from breast cancer patients (Groups A+B) with progesterone receptor negative and progesterone receptor positive tumours at the time of diagnosis. *P*=0.067 *vs* values of progesterone receptor negative patients.

The flow cytometric quantification of mature CEC showed significantly (*P*<0.01) elevated amounts of CEC in the patient samples (median: 5.7 CEC *μ*l^−1^ blood) at the time of diagnosis compared to the matched controls (median: 1.3 CEC *μ*l^−1^ blood) (Figure 1A). The analysis of CEC during the course of treatment showed that CEC were reduced under neoadjuvant treatment compared to pretreatment values (Figure 1B). This reduction was significant (*P*<0.05 and <0.01 respectively) in the blood samples taken before and one day after surgery. There was no direct correlation of CEC amounts with the concentration of the measured serum factors, including VEGF and sVCAM-1. The comparison of CEC levels with clinico-pathological data showed that the amounts of CEC did not differ between patients categorised into four clinical tumour stage (cT) groups. There was also no difference in the amounts of CEC when patients were subgrouped according to their tumour grading or Her2 receptor status. The classification according to the hormone receptor status showed significantly (*P*=0.025) increased CEC amounts in oestrogen receptor negative patients compared to receptor positive patients (Figure 1C). The progesterone receptor negative patients showed also increased CEC amounts when compared to the receptor positive patients, but this increase was not significant (*P*=0.067) (Figure 1D). Further, there was no correlation between the extent of CEC reduction during chemotherapy and the observed pathological tumour regression grade according to Sinn. Clinically, nine patients achieved a partial response. The clinical response of one patient was assessed as stable disease.

For the activated endothelial cells characterised by the expression of CD105 or CD106, we found no difference between patients and controls and also no changes during the course of anticancer treatment.

### Quantification of progenitor cells

**Figure 2:**
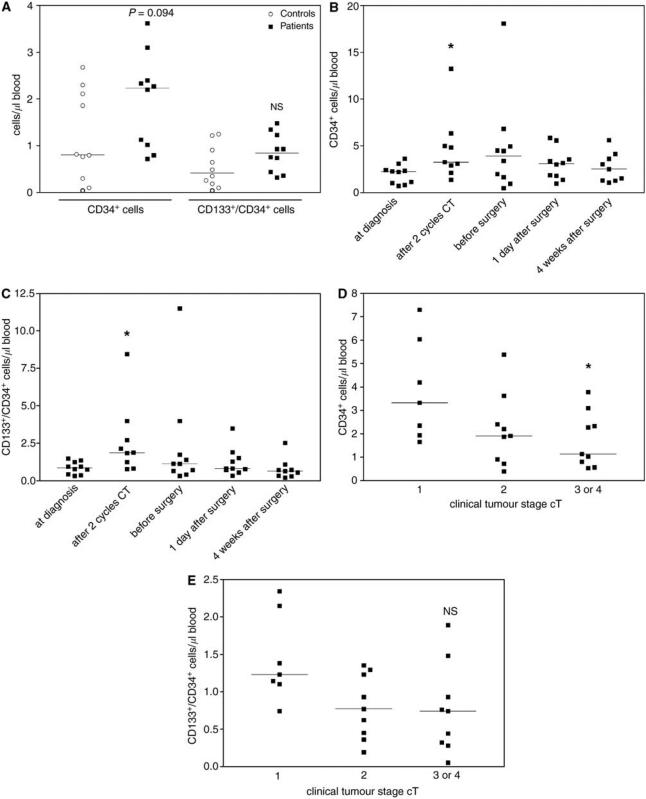
Flow cytometric quantification of CD34^+^ and CD34^+^/CD133^+^ progenitor cells. The amounts of CD34^+^ and CD34^+^/CD133^+^ progenitor cells were quantified in peripheral blood samples of breast cancer patients and matched controls by four colour flow cytometry analysis. (**A**) Comparison of the amounts of CD34^+^ and CD34^+^/CD133^+^ progenitor cells in blood samples from patients (Group A) at the time of diagnosis and matched controls. *P*=0.094 *vs* values of matched controls. (**B**) CD34^+^ levels during the course of neoadjuvant chemotherapy and subsequent surgery. ^*^P<0.05 *vs* values at time of diagnosis. (**C**) CD34^+^/CD133^+^ levels during the course of neoadjuvant chemotherapy and subsequent surgery. ^*^P<0.05 *vs* values at time of diagnosis. (**D**) Comparison of CD34^+^ amounts in blood samples from breast cancer patients (Groups A+B) with different clinical tumour stages at the time of diagnosis. ^*^P<0.05 *vs* values of patients with clinical tumour stage 1. (**E**) Comparison of CD34^+^/CD133^+^ amounts in blood samples from breast cancer patients (Groups A+B) with different clinical tumour stages at the time of diagnosis.

For the quantification of progenitor cells, we analysed the total population of CD34^+^ cells, which are the general haematopoietic stem cells, and the subpopulation of CD133^+^/CD34^+^ cells, the progenitor population that also contains the EPC. Early EPC characterised by the expression of CD34, CD133 and the VEGFR-2 (Flk-1, KDR) are present in the circulation at very low numbers and cannot be quantified by flow cytometry without previous enrichment procedure. But the more mature EPC, which are positive for CD34 and the VEGFR-2, could be measured in the blood samples. The amount of CD34^+^ cells was 2.2 cells *μ*l^−1^ blood (median) for the patients and 0.8 cells *μ*l^−1^ blood (median) for the controls (Figure 2A). This difference was not significant (*P*=0.094). The patients also displayed higher amounts of CD133^+^/CD34^+^ cells (median: 0.8 cells *μ*l^−1^ blood) when compared to the healthy controls (median: 0.4 cells *μ*l^−1^ blood), but this difference was not significant (Figure 2A). The amounts of CD34^+^ cells and CD133^+^/CD34^+^ cells were both significantly (*P*<0.05) increased after two cycles of chemotherapy compared to pretreatment values and these elevated levels normalised thereafter (Figure 2B and C).

Patients with clinical tumour stage (cT) 1 had significantly (*P*<0.05) higher amounts of CD34^+^ cells compared to patients with clinical tumour stage 3 or 4 (Figure 2D). This trend was also observed for the CD133^+^/CD34^+^ cells, but was not significant (*P*<0.1) (Figure 2E). There was no difference in the amounts of CD34^+^ cells and CD133^+^/CD34^+^ cells when patients were subgrouped according to their tumour grading, hormone receptor status or Her2 receptor status.

**Figure 3:**
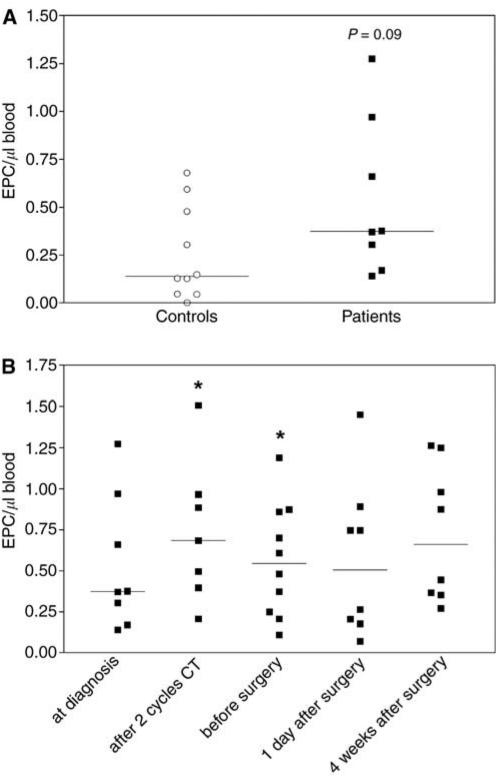
Flow cytometric quantification of EPC (CD34^+^/VEGFR-2^+^). The amounts of EPC were quantified in peripheral blood samples of breast cancer patients and matched controls by four colour flow cytometry analysis. (**A**) Comparison of the amounts of EPC in blood samples from patients at the time of diagnosis and matched controls. *P*=0.09 *vs* values of matched controls. (**B**) EPC levels during the course of neoadjuvant chemotherapy and subsequent surgery. ^*^P<0.05 *vs* values of matched controls.

The amount of circulating EPC (CD34^+^/VEGFR-2^+^) was 0.37 cells *μ*l^−1^ blood (median) for the patients at the time of diagnosis and 0.14 cells *μ*l^−1^ blood (median) for the matched controls (Figure 3A). This difference was not significant (*P*=0.09). After two cycles of chemotherapy, the amount of circulating EPC increased to 0.68 EPC *μ*l^−1^ blood (median). A closer analysis of this increase showed that in 50% of the patients EPC levels increased, whereas in the other half EPC amounts slightly decreased. The patients, who reacted to chemotherapy with an increase in EPC numbers, had low EPC amounts before treatment (median: 0.17 EPC *μ*l^−1^ blood) and their EPC counts increased after two cycles of chemotherapy to 0.97 EPC *μ*l^−1^ blood (median). The nonresponding patients already had 0.66 EPC *μ*l^−1^ blood (median) before treatment and EPC levels slightly decreased to 0.5 EPC *μ*l^−1^ blood (median) after chemotherapy. After two cycles of chemotherapy and before surgery, the amount of EPC was significantly increased (*P*=0.011 and 0.033 respectively) in comparison to the matched controls (Figure 3B).

### Measurement of angiogenesis-relevant serum factors

The concentration of serum factors that influence angiogenesis were determined by ELISA. The serum concentration of FGF basic was below the detection limit of the ELISA. For sVCAM-1 we found significantly (*P*<0.05) elevated levels in patients (median: 394 ng ml^−1^) compared to the controls (median: 253 ng ml^−1^). Serum levels of VEGF were higher in patients (median: 138 pg ml^−1^) than in controls (median: 92 pg ml^−1^), but this difference was not significant (*P*=0.1). The concentrations of erythropoietin, angiopoietin-2 and soluble endoglin did not differ significantly between patients and controls.

Oestrogen receptor positive patients showed elevated levels of endostatin (median: 121 ng ml^−1^) compared to oestrogen receptor negative patients (median: 104 ng ml^−1^). This difference was not significant (*P*=0.057). Also progesterone positive patients showed significantly (*P*=0.03) elevated levels of endostatin (median: 122 ng ml^−1^) compared to progesterone receptor negative patients (median: 103 ng ml^−1^). Concomitantly with the increase of CD34^+^ cells and CD133^+^/CD34^+^ cells, a significant increase of VEGF (*P*<0.05), erythropoietin (*P*<0.01) and angiopoietin-2 (*P*<0.05) and a significant decrease of soluble endoglin (*P*<0.01) were observed after two cycles of chemotherapy compared to pretherapeutic values (Table 2). The increase in the levels of VEGF, erythropoietin and angiopoietin-2 was followed by an increase of endostatin before surgery (*P*<0.01, Table 2). Erythropoietin remained elevated throughout the chemotherapy period, whereas angiopoietin-2 and VEGF returned to basal levels. Soluble endoglin remained decreased during the course of treatment.

## DISCUSSION

In the presented study we investigated the changes of mature CEC, EPC, their progenitor cells and angiogenesis-relevant growth factors during the course of anthracycline and/or taxane based chemotherapy and subsequent surgery. We could demonstrate that CEC levels are increased 4.4 times in primary breast cancer patients compared to matched healthy controls. This is consistent with previous findings where increased levels of CEC were described in patients with various cancer types in comparison to healthy controls (Mancuso *et al*, 2001; Beerepoot *et al*, 2004). We could further show that these elevated CEC levels decrease significantly after anthracycline/taxane based chemotherapy, but CEC levels did not reach the level of the healthy controls.

In a previous study it was described that plasma levels of sVCAM-1 and VEGF correlated with the amounts of CEC (Mancuso *et al*, 2001). We observed increased serum levels of sVCAM-1 and VEGF in patients compared to the matched controls, but we did not detect a direct correlation between any growth factor and the amounts of CEC. Since mature CEC are probably detached cells from activated or damaged vessels, their number could reflect the extent of tumour neovasculature. This is supported by the findings that in cancer patients elevated numbers of CEC are detected and that the CEC counts normalise when the tumour is removed by surgery or chemotherapy (Mancuso *et al*, 2001; Beerepoot *et al*, 2004). The number of CEC in tumour patients could be influenced by many factors as the localisation of the tumour and the extent of the tumour vasculature, the activation and proliferation status of the endothelial cells in the tumour area and also the activation of matrix metalloproteases that could facilitate the detachment of endothelial cells. Thus, it is not surprising that no direct correlation between a single growth factor and the CEC numbers was detected. Further, the number of CEC was not associated with clinical tumour grading or tumour size. This could again be explained by the complexity of factors that could influence the amounts of CEC.

In our small study, CEC levels at the time of diagnosis were significantly lower in oestrogen-receptor positive breast cancer patients compared to receptor negative patients. In parallel, oestrogen receptor positive patients showed elevated levels of endostatin. This was similarly true for progesterone positive patients (lowered CEC levels and elevated endostatin levels). Endostatin, an inhibitor of angiogenesis and tumour growth, is a fragment of the NC1 domain of collagen XVIII. It can be generated via proteolytic cleavage of collagen XVIII by cathepsin L or various other proteases (Felbor *et al*, 2000; Ferreras *et al*, 2000). An association of CEC numbers and endostatin levels with hormone-receptor status has not been described before and should be further analysed in studies with high patient numbers. This link could be part of the explanation for the better prognosis of hormone-receptor positive breast cancer. This aspect gains support by an observation that breast cancer patients with elevated endostatin levels following surgery showed a lower risk of relapse (Zhao *et al*, 2004). Further, a recent publication showed that endostatin dramatically inhibits endothelial cell migration, vascular morphogenesis and perivascular cell recruitment *in vivo* (Skovseth *et al*, 2005). These findings underscore the impact of endostatin on endothelial cells *in vivo* and sustain the hypothesis of a direct relationship between lowered CEC levels and elevated serum endostatin. We also observed a tendency to decreased angiopoietin-2 levels in hormone receptor positive patients underlining a relative antiangiogenic status in hormone-receptor positive breast cancer patients. A possible link between endostatin and oestrogen receptors could be the oestrogen-dependent regulation of proteases, for example, the induction of transcription of the lysosomal aspartyl protease cathepsin D by oestrogens (Cavailles *et al*, 1993). Thus, in oestrogen-receptor positive tumours, such proteases could be expressed leading to increased proteolytic release of endostatin.

Besides the impact of chemotherapy on CEC levels, we were also interested in the influence of this treatment on the amounts of EPC and their progenitor cells. Our results demonstrated that in the initial phase of chemotherapy, a general mobilisation of progenitor cells was induced. This is a well-known phenomenon observed during various chemotherapy regimens (Schwartzberg *et al*, 1992). The induction was marked on a cellular level by elevated amounts of CD34^+^ stem cells, CD34^+^/CD133^+^ progenitor cells and circulating EPC and was accompanied by increased levels of VEGF, angiopoietin-2 and erythropoietin. Of the multitude of growth factors that regulate physiological and pathological angiogenesis, VEGF is believed to be the most important. VEGF is a potent mitogen for vascular endothelial cells and it is also essential for the mobilisation of bone-marrow-derived endothelial precursors (Asahara *et al*, 1999a). Concurrently, erythropoietin is not only mobilising the CD34^+^ stem cells, it also increases the number of functionally active EPC as it was shown in humans after administration of recombinant erythropoietin (Bahlmann *et al*, 2004). In tumour patients with relative high amounts of EPC, chemotherapy did not induce a further increase in this population, whereas in patients with low EPC numbers chemotherapy induced a 5.7-fold increase in circulating EPC amounts. The increased amounts of EPC, the mobilisation of the progenitor pool from which the EPC originate and increased proangiogenic serum factors reflect an increased angiogenic potential induced by chemotherapy. This first observation from our small study raises the question if this initial proangiogenic state is of clinical relevance in cancer disease and should be covered by a concomitant antiangiogenic therapy. This question seems particularly important regarding the vessel-forming potential of EPC: EPC have a high proliferative and regenerative potential, they can home to sites of neoangiogenesis and differentiate into mature EC in response to angiogenic growth factors (Asahara *et al*, 1997, 1999b; Takahashi *et al*, 1999; Kalka *et al*, 2000; Lin *et al*, 2000; Murohara *et al*, 2000). Bone marrow derived haemangiopoetic progenitor cells can fully support tumour growth (Peichev *et al*, 2000) and contribute to tumour neovasculature (Davidoff *et al*, 2001), as demonstrated in bone marrow transplant models. Based on these aspects, an increase in EPC or the progenitor cells that could differentiate into EPC may pose a risk for the patients since these cells could contribute to tumour neovascularisation and thus could increase the risk of relapse. Therefore, it seems worthwhile to design clinical trials combining chemotherapy with emerging antiangiogenic drugs to explore the impact of the initial prohaemangiogenic status induced by chemotherapy.

In conclusion, we could show that both mature CEC and EPC are influenced by chemotherapy: CEC numbers declined during chemotherapy, whereas EPC and their progenitor pool were mobilised. For the assessment of the angiogenic status and for evaluation of the effectiveness of antiangiogenic therapies, it is important to consider both populations (together with relevant angiogenic serum factors), since both of these populations could contribute to neovascularisation.

## Figures and Tables

**Table 1:** Characterisation of patients analysed in this study

**Patient**	**Age (years)**	**cT**	**ER (+/−)**	**PR (+/−)**	**HER2 (+/−)**	**Tumour grading**	**Therapy**	**Regression grade after Sinn**
*(A) Patients Group A*								
1	38	3	+	−	ND	ND	ET (6 cycles)	3
2	40	2	−	−	+	3	CEF (6 cycles)	1
3	52	3	−	−	+	3	CEF (4 cycles), trastuzumab/paclitaxel (6 cycles)	4
4	61	2	−	−	+	3	ET (5 cycles)	0
5	56	2	+	+	−	2	FEC (6 cycles)	1
6	54	3	+	+	−	3	CEF (5 cycles)	1
7	48	4	+	+	−	2	CEF (6 cycles)	2
8	42	4	−	−	+	3	CEF (5 cycles), trastuzumab/paclitaxel (4 cycles)	0
9	43	4	+	+	+	3	ET (6 cycles)	3
10	59	2	+	+	−	3	CEF (5 cycles)	1
								
								
*(B) Patients Group B*								
1	37	2	−	−	−	3		
2	55	1	+	+	−	2		
3	69	1	+	+	−	1		
4	60	1	+	+	+	1		
5	54	1	+	+	−	1		
6	66	2	+	+	ND	3		
7	69	1	+	+	ND	NA (pT=1 mm)		
8	44	1	+	+	−	2		
9	61	1	−	−	+	3		
10	67	Tis (DCIS)	+	+	ND	NA		
11	67	2	+	+	+	2		
12	39	3	+	+	−	2		
13	41	3	+	+	+	2		
14	71	3	−	−	ND	3		
15	39	2	+	+	−	3		
16	61	2	+	+	−	1		

Group A: Blood samples of these patients were analysed during the course of neoadjuvant chemotherapy and subsequent surgery. (B) Group B: Blood samples of these patients were analysed at the time of diagnosis.

cT: primary tumour size; ER: oestrogen receptor expression; PR: progesterone receptor expression; HER2: overexpression of HER-2/neu; ET: epirubicin/docetaxel; CEF: cyclophosphamide/epirubicin/5-fluorouracil; FEC: 5-fluorouracil/epirubicin/cyclophosphamide; ND: not determined; NA: not applicable, pT: pathological tumour size.

**Table 2:** Measurement of serum VEGF, erythropoietin, angiopoietin-2, soluble endoglin and endostatin

	**At diagnosis**	**After 2 cycles chemotherapy**	**Before surgery**	**1 day after surgery**	**4 weeks after surgery**
VEGF (pg ml^−1^)	138 (106/170)	167^*^ (99/257)	147 (106/230)	161 (116/199)	164 (104/274)
Erythropoietin (mIU ml^−1^)	14.5 (9.9/17.9)	23.4^*^ (16.4/31.6)	20.3^**^ (12.8/27)	38.8^*^ (25.1/53.4)	23.9^***^ (21.4/28.7)
Angiopoietin-2 (pg ml^−1^)	2317 (1682/2651)	2935^*^ (1874/3309)	2280 (1874/3309)	2474 (2012/2760)	2370 (2169/2656)
Endoglin (ng ml^−1^)	4.4 (4.1/5)	4.2^**^ (3.8/4.5)	4.3 *P*=0.066 (4/4.6)	3.7^***^ (3.4/4)	4.3 (4.1/5)
Endostatin (ng ml^−1^)	109 (98/119)	119 (91/141)	122^*^ (112/144)	110 (97/124)	119 (113/127)

The concentrations of VEGF, erythropoietin, angiopoietin-2, soluble endoglin and endostatin were quantified by ELISA in serum samples of breast cancer patients during the course of neoadjuvant chemotherapy and subsequent surgery. In the table, median values (25% percentile/75% percentile) are given. ^*^P<0.05, ^**^P<0.01, ^***^P<0.001 *vs* values at time of diagnosis.
