# Supplementary figures and images for: Circulating endothelial cells and angiogenic serum factors during neoadjuvant chemotherapy of primary breast cancer
Source: Br J Cancer. 2006 Jan 31;94(4):524–31. doi: 10.1038/sj.bjc.6602952 (PMC2361171; doi:10.1038/sj.bjc.6602952)

# Supplementary Figure 1

A) Mature CEC

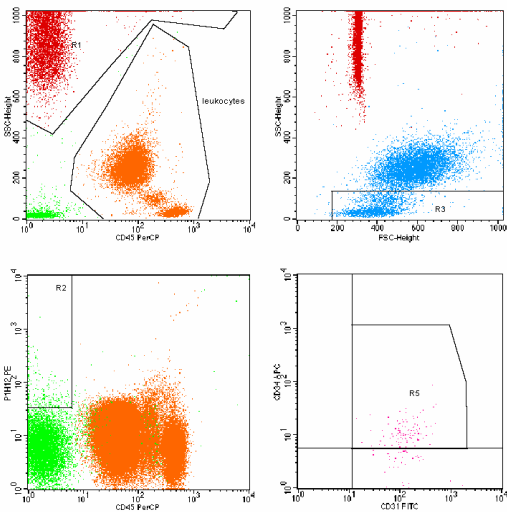

B) Activated CEC

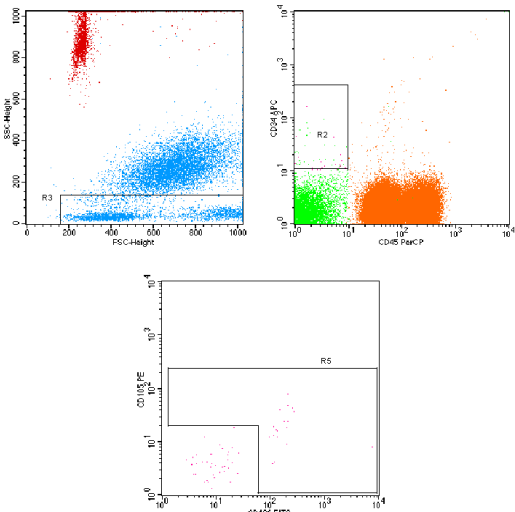

C) CD34 and CD133/CD34-positive cells

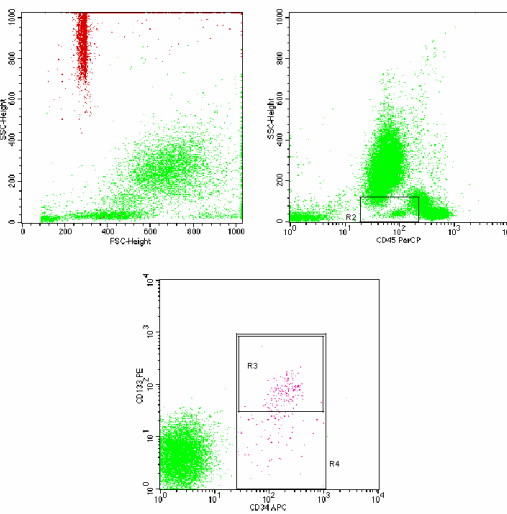

D) EPC

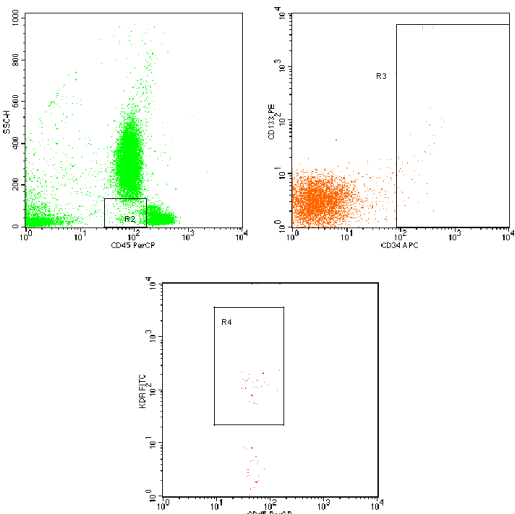

Supplement: Supplementary Figure 1 [file 94-6602952x1.pdf]
